# Personal support and expressions of care for pregnant women in Soweto, South Africa

**DOI:** 10.1080/16549716.2017.1363454

**Published:** 2017-09-06

**Authors:** Langelihle Mlotshwa, Lenore Manderson, Sonja Merten

**Affiliations:** ^a^ Department of Epidemiology and Public Health, Swiss Tropical Public Health Institute, Basel, Switzerland; ^b^ Department of Epidemiology and Public Health, University of Basel, Basel, Switzerland; ^c^ Division of Epidemiology and Biostatistics, School of Public Health, Faculty of Health Sciences, University of the Witwatersrand, Johannesburg, South Africa; ^d^ School of Public Health, Faculty of Health Sciences, University of the Witwatersrand, Johannesburg, South Africa

**Keywords:** Care, interventions, masculinity, maternal health, pregnancy, South Africa

## Abstract

**Background**: Pregnancy is life changing, making great demands on women to adapt physically, psychologically, and socially. Social relationships and the support that flow from these provide a critical role in managing health problems in pregnancy. Isolation and lack of care, in contrast, may lead women to experience increased distress during this time.

**Objective**: This study aimed to explore South African women's perception and experience of care and support in pregnancy.

**Methods**: A life history approach was employed to explore women’s experiences of pregnancy and sexual behaviour, with each participant encouraged to narrate important life events from her own perspective. We drew on narrative interviews with 15 pregnant women, conducted between July and October 2015, in which we explored questions regarding pregnancy planning and the provision and receipt of care. A thematic approach was employed to code and analyse the data.

**Results**: Themes that emerged from the interviews showed that participants gained a sense of stability in their lives when they had support in their pregnancy, especially when dealing with challenging situations. This support came variously from family, friends, and social networks. Overall, those participants who mentioned the most support, and its diversity across different groups, reported a better experience of pregnancy.

**Conclusions**: Women emphasised the importance of social and emotional support in pregnancy. Understanding women’s experiences can assist in making pregnancy less overwhelming, and can add to a woman’s ability to deal with different challenges before and after the arrival of the new baby.

## Background

Pregnancy is life changing, making great demands on women to adapt physically, psychologically, and socially [1]. It is a transition in life which brings many challenges [1,2], and for this reason, the care and support extended to women can impact greatly on their lives and contribute to how they experience the pregnancy [1–4]. Social relationships and support, both formal and informal, play critical roles in managing health and personal problems in pregnancy [5–7]. Reblin and Uchino [2] differentiate between tangible care and support, in the form of money, shelter, clothing, and food, for example; and intangible care and support, including emotional, psychosocial, and perceived or received support. The range of material, contextual, and interpersonal factors that are therefore included reflect the complex phenomena that impact on women [1,2]. These personal, social, and cultural factors vary in different contexts.

In South Africa, more than 50% of children are born out of wedlock and grow up in single-parent households, of which two-thirds are headed by women [8–11]. As a result, significant numbers of women carry, give birth to, and care for their infants without the support of the biological father [12]. On learning of their pregnancy, women must negotiate their relationship with the father of the unborn child, with their own family, and with others. In some cases, the social repercussions of an unintended pregnancy may leave a woman with little or no care from those from whom she might have expected support, with impacts on her health and well-being [13,14]. Accordingly, many women need to make various compromises in their own care and in sustaining the household, including in relation to new intimate relationships established when they are pregnant or when the baby is very young [15,16]. At the same time, as we illustrate below, many women invest in the relationship related to their pregnancies, and retain the hope that these men will accept the pregnancy and commit to shared parenting.

In South Africa, research on social dimensions of unexpected and unplanned pregnancies has tended to concentrate on women who, despite variation in different urban and rural settings, tend to be marginalised [17–21]. The incidence of human immunodeficiency virus (HIV) appears to be higher among young pregnant women than their non-pregnant counterparts [22,23], suggesting an association with the irregular use of protection; communities with high rates of teenage pregnancy also have concomitantly high poverty rates [24,25]. The concurrence of poverty, HIV, and unplanned pregnancy affects older women as well, often leading to a toxic environment of depression and violence [13,14,26]. These combined factors impact on women’s capacity, and that of their families, to manage the material as well as social and interpersonal challenges associated with pregnancy. In a study in KwaZulu Natal on social support and pregnancy, women were most likely to disclose their newly diagnosed status as HIV positive when they felt that they could live at home and feel safe and supported [27]. South African women generally attend at least one antenatal visit and give birth with a skilled birth attendant [28], and consequently antenatal care (ANC) is a potentially important point of intervention, hence our focus.

In this article, we define care and support received as the material and practical assistance and emotional support given to women throughout pregnancy, primarily through affective ties. The support of the partner plays a crucial role [1,4,29], including in a woman’s decision to keep the pregnancy, as described in various studies around Africa [16,21,27,30]. We explore what pregnant women perceive and experience as care and support, with data illustrating the significance for pregnant women of having a network of people to enable them to cope with different stressors that arise during pregnancy.

## Methods

### Study design and setting

The study on which we draw was undertaken with women presenting for ANC at Chris Hani Baragwanath Hospital, in Soweto, Johannesburg, South Africa. The study was nested in a larger research project on incident HIV in pregnant women and sexual risk behaviours and practices in urban South Africa (Pregnancy and HIV Study). This, in turn, was nested in a larger prospective cohort study, the Soweto First 1000 Days Study (S1000) [31], which followed women and their infants for up to 24 months after delivery at Chris Hani Baragwanath Hospital. S1000 registers women into the study before or at 20 weeks’ gestation, regardless of their HIV infection status, and so includes women who are HIV positive at the time of conception. In contrast, the Pregnancy and HIV Study selected women who were HIV negative at entry. Women were tested for HIV 12 weeks after the initial test and subsequently at each antenatal visit until delivery. Women in the study we report here were recruited from the Pregnancy and HIV Study, and so were all HIV negative at the time of our first interactions. A life history approach was employed with those who agreed to participate, as elaborated below, to explore their experiences of pregnancy and sexual behaviour in pregnancy, with each woman encouraged to narrate important life events from her own perspective [32]. Over time, a number of these women seroconverted, either because the original test failed to identify they were already infected, or because of an infection acquired during pregnancy. In this article, we draw on narrative interviews with 15 pregnant women recruited when they first enrolled into the larger study and were HIV negative. No participants acquired an HIV infection during the course of our study. This had the advantage of allowing us to focus on tensions around the pregnancy and effective relationships, independent of HIV status.

### Data collection

Multiple interviews were conducted during women’s pregnancies between July and October 2015, at a place where both the researcher and the participants were comfortable, that is, the hospital or the home of a given participant. An initial interview at the end of the first trimester provided us with a general understanding of the woman’s life history and enabled the interviewer (first author) and the participant to establish a relationship of relative trust and comfort [33,34]. A second interview, conducted at the end of the third trimester, helped to clarify issues that emerged in the first interview while focusing on the woman’s current intimate relationship(s) and sexual behaviour. Final interviews, not included here, were conducted after delivery to explore changes in sexual behaviour and practices; by this time, two women had HIV seroconverted. All interviews were conducted by the first author in English, seSotho or isiZulu, languages in which she is fluent. Often participants moved between these languages, as is characteristic in the study area. Each interview took between 20 and 96 min, and all were audiotaped, transcribed, and translated verbatim (into English, as needed). All participants were aged 18 years and above.

### Data analysis

A thematic approach to developing primary codes, subordinate codes, and categories was used to guide the analysis of the data, with interviews analysed inductively [34]. Reflecting one of the strengths of this approach, data collection and analysis occurred simultaneously to inform subsequent data collection [34,35]. Initially, each narrative was read and reread and coded openly for content. With the continued rereading of the narratives, theoretical coding was introduced as a second step, with codes grouped into emerging themes. The emerging themes were developed through analysing their salience within and among interview accounts. Coding was validated by the coauthors, and, when codes were irregular or inconsistent, consensus was reached after reanalysing the codes. Table A1 in the Appendix provides an example of how these categories were formulated.

## Results

### Profile of participants

All 15 participants were aged between 25 and 40 years; the majority (12) had completed secondary school and some (four) had then gained post-secondary school qualifications or vocational certificates. Nearly all women (13) reported having a partner at the time of the first interview, although five of these women did not live with their partners. Four were customarily married. Four reported that their partners were known by their families and they were cohabiting, but no formal arrangement had been made for compensation (*inhlawulo*) for the ex-nuptial pregnancy, nor had bride price (*lobola*) been paid. The other two women were not sure how to define their relationships, other than as ‘complicated’. The length of time of the relationship with the genitor of the current pregnancy ranged from less than 1 year to more than 10 years. Almost all participants (14) reported that they had not planned the pregnancy. Only one participant was pregnant for the first time; for 14, the current pregnancy was the second or third; seven reported miscarriage of the previous pregnancy. Four of the participants were mothers to older children, all by a different father. Eight of the participants had migrated to Soweto to marry, to look for employment, or for family reunions, but most had limited family in the area at the time of the interview. Nine women had some paid employment; the rest had no source of independent economic support.

### Informal patterns of support and care

Participants reported that they enjoyed a sense of stability in their lives, especially when dealing with challenging situations, through the presence of immediate family, including partner, mother, sister, and/or uncle. At the same time, they differentiated kin as offering different kinds of support and care during their pregnancy, and identified other people who contributed substantially to their well-being. As women illustrated in their narrative accounts, these experiences of care were mainly centred on their partner, then on immediate family members, friends, and others from various social networks and organisations. We present these below, in ways that reflect the relative significance of different individuals and relationships, and the variability of families, households, and other support structures in contemporary South Africa [36].

### Partner intimacy and involvement

About 40% of all households in South Africa are headed by women [12,16,37], reflecting historic patterns of male labour migration, HIV-related mortality, and more recent trends in female migration and family formation outside marriage [12]. Only around one-third of South Africans over the age of 20 are registered as married; divorce is common, with the rise of 8.6% from 2012 to 2013 reflecting a continuing trend [38]. Female-headed households are supported by access to different social services and systems of support, for example, income grants and housing, and this provides women with some financial support [16]. Yet despite demographic trends that indicate the prevalence of low marriage rates, short-lived unions, and female-headed households, all participants spoke about the importance of having a supportive male partner during their pregnancy and in the future, and emphasised the value of dual parenting. Many felt that the support from their partner was the most important source of support they could receive, as the two of them ‘needed to be in it together’. Women emphasised the affective nature of such support, and often reiterated that even if a partner supported the woman financially or in other ways, if he did not show affection or closeness in the relationship, he was considered to be unsupportive:

‘I need support from the father of my child; it is the most important support I need. But I do not get it. This bothers me a lot, even if he is there but he is not giving me the support I feel I deserve [crying] … I need him to be there for me, emotionally I need to know that we are in it together … The support I received with our first child was good, but now there is none. When I raise it with him, he tells me that I am complaining and I should not be complaining [continues crying].’ (Busi, 27)

Some participants spoke about the possibilities of their partners having other intimate relationships, which they interpreted as indicative of the man’s lack of emotional commitment. However, they also noted that it was not uncommon for men to have outside relationships, and to some extent, this was ‘socially acceptable’. Although women recounted their distress when the men in their lives had relationships with other women, many insisted that they were willing to accommodate this in order to preserve their relationship as primary, for their own sake because of the pregnancy, and so for their future child. At the same time, most women spoke of their fear of HIV; Lillian (38) told her partner simply that if he decided to have extramarital relationships, he needed to use a condom as they were not using a condom in their own relationship. In a country where 29.7% women are HIV positive at entry into ANC [39], their concern that they might become infected was legitimate:

‘Yes, it is possible for him to have [other sexual relationships]. For example I’m sitting with you right now, I am saying he is at work, but it’s possible he is elsewhere doing his own things. I mean you cannot trust someone 100% but what I know is, I told him that if it happens! If it happens, that you [the partner] are seeing someone else outside of our marriage and you are having sex, please use a condom. No one should bring death [through HIV] into this home ever. We know you are the one as the man who will fail to have self-discipline or self-control, and so if you decide to go out wherever, please use a condom.’ (Lillian, 38)

While this might suggest that women were accommodating, their responses indicated that they were worried and sometimes distressed that their partners might have other sexual partners, and that they may have to deal with HIV in the ‘home’, that is, within their relationship. Women did not want to acquire HIV under any circumstances.

In explaining or seeking to understand men’s relationships with them and in relation to other women, participants drew a link between men not wanting the pregnancy and their lack of concern and care for the woman during pregnancy. Unplanned pregnancies, women explained, precipitated men’s disengagement and detachment:

‘I don’t think he is even part of this pregnancy. He is just not interested, there is no support from him … I actually think it’s because of the pregnancy, the fact that I got pregnant too soon. Maybe he is not ready for this. However, even when all this is going on, you find that if I need anything he will give me the money for it. If I say I need something, he has no problem with that, but the emotional side to the relationship is dead. I mean really, being in a relationship and there is no emotional attachment, honestly, I don’t want to lie, it is disturbing, it really is.’ (Rachel, 27)

Women who still had a strong emotional attachment with their partner spoke of constraints of the relationship, including with regard to their pregnancy and its course. Some women reflected on the value of visiting the ANC clinic together, but for the most part they accepted that their partner did not understand the importance of a visit to the ANC, was not interested in it, and was not prepared or able to go with them. Women tended to interpret this in the context of a difference in background:

‘I would like to attend ANC with him. It would be really good for us both, so that we experience everything together. But I need to also accept that we come from different backgrounds so the way I take things could be different from him.’ (Rhirandzu, 32)

On the other hand, some participants received extensive support and care from their partners, and they found considerable comfort in having someone on whom they could depend:

‘He treats me well, we have been together for about 10 years now and he respects me. As the woman in his life, I cannot complain really. He loves me and takes care of all my needs. I am really happy. He even takes care of not only his children but my children. I don’t want to lie he is good to me. I know sometimes you will say someone is good then he disappoints you, but he is good to me.’ (Boniwe, 39)

### Immediate family involvement

While participants emphasised the importance of support from their partner, strong family support was also considered important, particularly by women whose partner had retreated or gave them limited support. Most of the time the person involved was female kin – a sister or aunt – although uncles were also mentioned as assisting them during their pregnancy. Mostly participants spoke about emotional, physical, and monetary assistance from female support, while male support was mainly linked to financial contributions.

‘Emotionally my mom does this for me [gives me support]. She is there for me even when I start to complain and say, ‘Ahhhhh, my back hurts so much’, she is the one who will calm me down. She will say, ‘no, do not worry, it will all be okay’. So yes my mom.’ (Daisy, 30)

‘My sisters, yes [they are there for me] but there is really nothing much [financially] that they do, they are my friends, so we talk and yah, that’s it. They are the ones I consider my friends. They give me good advice, just talking to them is good.’ (Ntombi, 25)

‘My uncles are those types of people who do not show outward emotion. They are supporting me in other ways, for example money. They are just angry because the father of the child does not want to take responsibility for his actions (the pregnancy); this is what angers them.’ (Hazel, 33)

### Support from in-laws

Some participants reported having good relationships with their partner’s parents or other family members. Sometimes when the couples were experiencing interpersonal difficulties, members of their partner’s family would call or would ask the participant to come over, so that they could make sure they were fine. In some cases, they would provide the woman with material support as needed, for example, with food to eat.

‘When they [partner’s sister and mother] see I have not visited in a long time they call to check on me … they ask me if I need anything and if everything is going on well with the baby.’ (Rachel, 27)

‘Although we are not together with the father of my child … his sister is really good to me, she gives me so much support. I don’t know whether it’s because we work together but she’s really good to me.’ (Hazel, 33)

In other cases, however, the relationship between a pregnant woman and her partner’s kin was not warm, and this brought considerable emotional distress to the woman or to the couple. Some participants felt that this lack of concern, acceptance, and affection caused conflict or unsettled their relationship with their partner:

‘What I think the main challenge or problem is just our parents, this is what most the times is hurting our relationship … I think the problem comes with his side of the family, mainly. I don’t think they like me, not me as a person but as long as there is anyone involved with their child emotionally they will not be happy about it.’ (Ntombi, 25)

### Support from friends

Not all participants had friends on whom they could depend. However, women who described themselves as single, or who had partners who lived at a distance from them, emphasised the value of regular support from friends:

‘My friends are there. I remember the last time I was admitted in hospital they are the ones who made sure they took me to hospital. My partner he was not even there, he works away from home. They called him and told him that he should not worry; if anything needed his attention they would let him know. So I have good friends who are able to take care of me.’ (Rhirandzu, 32)

Having a circle of friends on whom they could depend, who were willing to assist them during their pregnancy and beyond it, was critical when women lacked support from their own and/or their partner’s families. The care that they received from friends ranged from psychosocial support, being able to talk to someone about issues that were personally challenging and receiving advice on how best to cope with these issues, to transport when going to the hospital or elsewhere:

‘Yes, I have friends, we talk about everything if I’m stressed we talk about it all, they come to my house and fetch me and I visit them at their houses and we pray together things like that, they are the only ones who are my support … My sister and I we do not talk about personal issues. That is just how our relationship is like.’ (Pretty, 30)

### The church as a site of support

Those participants who reported having a religious affiliation – the majority – often spoke of how, once pregnant, they had stopped going to church. This was usually not because of ex-nuptial pregnancy, but because it was a long distance to walk or to reach by bus or taxi when they were tired, or because, they said, they had just become lazy. In their stories, they reiterated the importance of this aspect of their life, particularly those who had few or no kin living with them in Johannesburg. The church served as a support structure that, women felt, would be always there to assist them in times of need. Furthermore, although some women had stopped attending services, they still often received some care from church members. In particular, older women often spent time with them during pregnancy and postnatally, advising them about pregnancy, birth, and infant care, but also talking to them about faith and church expectations.

‘At my church you are not supposed to fall pregnant until you have worn your white dress, or without you having been married. … So now I am not active in that anymore, until they take me back, that old lady needs to say if I am ready or not. The lady who talks to you and helps you out in pregnancy she needs to say you are ready or not. Now what they have done is assigned a lady in church to look after me through this process of ‘sin’ that I am in, so that after it’s all over I am able to go back to church … The thing is according to them the lady helps in the process of becoming okay something like that or me being ready in the eyes of the church that I can go back to doing my normal activities in the church.’ (Daisy, 30)

‘At church they are always there for you, especially the older ladies in the church, who come and check up on you and the baby if you are doing okay. They offer advice that is important, for example they tell us that the baby should be moving when you eat, when you are hungry it is important to have the baby move all the time, they tell us we should rub the tummy with Vaseline [petroleum jelly] to make sure the baby is always moving. If you do not feel any movement then it’s important to go to the doctor immediately.’ (Rhirandzu, 32)

Some participants who were unmarried reported that their church disapproved of them being unmarried and pregnant, so they had decided to stay away through embarrassment and to avoid criticism:

‘Like right now, because I am not married, I cannot go to church, they say that when you are not married, how can you get pregnant? So I cannot go to church. I cannot wear my church uniform. At church you cannot be seen pregnant when you are not married, but if I was married, it would be okay, there would not be any problems.’ (Rachel, 27)

Some further explained that they were protecting their own reputation by not going to church; in this way they would not be judged because they were unmarried and pregnant. These values were often shared by members of participants’ families. Family members believed that a man should marry a woman before parenting by paying bride price *(lobola)*, or at least paying damages *(inhlawulo)* to show their remorse of the ‘unintended’ pregnancy.

Overall, those participants who mentioned the most support, in terms of numbers, range of supportive actions, and diversity across different groups, reported better experiences of pregnancy. They reflected that they did not need to worry about many things, as they were taken care of by others. This reduced their levels of distress. Women with little support often complained about their relative isolation, and felt hurt and resentful about the absence of care. They reiterated the difficulty of being pregnant and of trying to meet their various material, practical, health, and emotional needs. Participants also mentioned their need for places where they could talk about issues that bothered them, or places that provided them with a safe haven. Many reiterated that the research clinic fulfilled this function, enabling them to talk about personal and relationship concerns. Women stated that the ‘nurses’ – any staff member of the research clinic – made them feel comfortable, and provided them with an opportunity to talk through and sometimes cry about their problems. They contrasted this with ordinary antenatal clinics, which were crowded and where clinic staff had neither the time to talk confidentially with them nor the willingness to engage with them about social and interpersonal problems.

## Discussion

While attention has focused on physical changes and discomfort during pregnancy, far less has been paid to women’s emotional, economic, and social well-being [40], especially in African settings [but see 13,14,30,41, 42]. In women’s narratives, a dominant theme was that pregnant women at times felt vulnerable and emotionally alone, and sometimes were unable to identify what was bothering them or had no one with whom to discuss what was going on in their lives [40,43]. Pregnant women hoped for care that met their personal as well as practical needs, and this might be from a person, a social organisation, a public institution, or a combination of all entities. At the same time, while women emphasised that support came from diverse quarters, they thought that the partner – the biological father – should be present to support and care for them throughout their pregnancy [44]. Some women felt that their partners did not understand what kinds of support and care they needed during pregnancy; they explained this in terms of conventional ideas of masculinity that conflated ideas of what a man is ‘supposed’ to do: take care of everything financially, but not by providing emotional support [45–47]. In South Africa in particular, women characterised masculinity in terms of men being providers in the home or within the relationship [48–51]. A strong masculinist ideology means that a man may be reluctant to show care and concern for a woman in pregnancy, by attending the ANC clinic, for instance, as this could be seen as weak and a mark of femininity [48]. Yet while many of the women reported a need to have their partner show care by providing for them (housing, cash), they also emphasised their desire for emotional care, for their partner to show his affection for them, including his involvement in and implied commitment to the pregnancy, through his physical presence as much as his financial support. Furthermore, while women spoke of dominant representations of African men’s sexuality in relation to multiple partnerships [47,49,50,52], they complied primarily because they felt that this would reduce their risk of being abandoned.

An increasing number of couples are delaying marriage in South Africa, for reasons that include the high costs of marriage, the payment of bride price and rituals associated with it, and the reluctance or inability of men to take care of a family [47,53]. Age at marriage in South Africa is between 25 and 29 years for women and 30 and 34 years for men [53], which is relatively high globally and compared with other countries in Africa. Ex-nuptial childbearing is common, accounting for almost 60% of all births in South Africa, among the highest in the world [45,47]. Furthermore, as noted above, female-headed households are becoming increasingly common, with households often constituted of a woman, her children, and grandchildren, and with women’s employment and state grants providing the financial resources to meet everyday household expenses. Yet in this current study, pregnant women in relationships were seeking more than material or instrumental support. With limited emotional support, pregnant women often were in strained relationships within and beyond the household, and loneliness and despondency were common for those who feared being left alone to care for the expected baby.

Women with more sources of support, including a reliable and loving partner and siblings, reported less stress than those with less support, replicating the findings of similar studies [31,40,46]. Social organisations like the church also played an important role in the coping strategies of some women. In some churches, the emphasis on abstinence before marriage meant that some pregnant women felt ashamed and withdrew, and the stigma of pre-marital sex and ex-nuptial pregnancy kept them from this avenue of support and care. Some women described how, after church members found out that they were pregnant, they were required to undergo a period of cleansing, after which they could resume their duties as a member. This sometimes hindered assistance from the church during pregnancy. However, other churches made women feel welcome and congregants shared their everyday concerns.

Financial challenges [44] and marital status [46] were issues that we anticipated from our participants and other studies [14,41,54]. Many of the women interviewed had limited sources of income to sustain them, but although they spoke about their need for money, this was not the primary support that they identified they needed from their relationships and in pregnancy. In women’s accounts, the need for personal support dominated. When the relationship was not working with the partner, pregnant women identified their need for a safe and confidential space to talk about these challenges. Personal matters were sometimes better and more easily discussed with other people and not with family members, so avoiding the fear of stigma and the risk of disclosure.

### Limitations

The study was qualitative and was localised in an urban area in South Africa. Women may have overstated their concerns in the hope that we might speak to their partners with regard to any concerns that they had, as we had sought their approval to also interview the men.

## Conclusion

For women throughout pregnancy, social and emotional support was vital. Pregnant women were eager to talk about issues that bothered them, and they seemed to struggle to find spaces where they could talk about these issues. Different people were identified as being available and able to provide of this kind support and care, and most of the time this ensured a smooth or less difficult time through pregnancy. On the other hand, women emphasised the value of a partner who was involved in the pregnancy. However, he was not always available, and in other cases, the relationship with the partner was fragile or tenuous.

Policy reforms in South Africa may need to address the importance of social support for pregnant women, to manage the emotional and personal difficulties that they experience during pregnancy [55]. This may be through support groups organised at hospitals and clinics, where women could share personal concerns, although women worried that in this context, they could be the subject of gossip. Self-help groups and mentoring programmes for women during pregnancy as well as after delivery, such as the Philani intervention programme in Khayelitsha Cape Town [56], may be a way for pregnant women to team up and talk about the different challenges they face, and to receive practical support, so reducing anxiety and depression. As many women have strong religious affiliations, they may also find support through self-help groups in church settings, with mentorship and encouragement by older women to help them better cope with pregnancy and early infant care. This may be particularly important for young women who do not have a family or who have a stressful relationship with family members. Understanding these experiences can assist in making a pregnancy less overwhelming, and can contribute to a woman’s ability to deal with various practical and relational challenges both before and after the arrival of the new baby.

## Paper context

Pregnancy can be overwhelming and life changing, demanding change from women physically, emotionally, psychologically, and socially. This paper is set in an urban African context were many children grow up without their biological fathers. The paper assists in understanding what women perceive as support during pregnancy. Understanding these experiences can add to a woman’s ability to deal with different challenges before and after the arrival of the new baby in similar settings.

## Appendix

**Table A1.** Examples of coding.

Interview text	Codes	Subordinate codes	Categories
I need support from the father of my child; it is the most important support I need. But I do not get it. This bothers me a lot, even if he is there but he is not giving me the support I feel I deserve [crying] …	Care during pregnancy	Support and affection	Partner intimacy and involvement
Emotionally my mom does this for me [gives me support]. She is there for me even when I start to complain and say, ‘Ahhhhh, my back hurts so much’, she is the one who will calm me down. She will say, ‘no, do not worry, it will all be okay’.	Emotional care by mothers, sisters, aunties	Responsiveness during pregnancy	Immediate family involvement
Although we are not together with the father of my child … his sister is really good to me, she gives me so much support. I don’t know whether it’s because we work together but she’s really good to me.	External assistance	In-laws’ presence	Support from in-laws
Now there is this thing that when you fall pregnant without going through the right path you get cut off from the congregation. You get cut off such that even when you pray in church the person next to you should not hear you, even when you sing he or she should not hear you.	Church and how they assist during pregnancy	External groups caring	Social organisation
